# Effects of Noticing Quacks

**Published:** 1868-09

**Authors:** T. A. McGraw


### Effects of Noticing Quacks.

It is a question, worthy of consideration, how much of the success of systems of quackery and of individual quacks, is due to the notice taken of them by the regular profession. Every righteous instinct urges honest medical men to expose and denounce the charlatans who practice on the credulity of the public and bring reproach on our profession. And yet it may be the part of wisdom to curb our natural instincts when we find that opposition serves only to advertise the offenders. It is better to witness evils which we cannot cure, in silence, than to aggravate them by injudicious expressions of hostility. We cannot escape the conviction that the well meant but violent and impolitic opposition of some of our medical brethren to certain forms of quackery has done much to enlist the sympathies of the public in favor of the quacks. Folly, in itself ridiculous and short-lived, becomes dignified into a system which will command the support of numerous and enthusiastic converts, if made the object of an injudicious persecution. In most people the feelings of sympathy are more powerful than the dictates of judgment, and to know that a man suffers at the hands of other men, though his sufferings may be the legitimate fruits of his own wickedness, is sufficient to prejudice many in his favor. There are consequently many men, especially in the medical profession, who are martyrs by trade, and deliberately seek to arouse an antagonism which may place them in the eyes of the community in the enviable position of persecuted individuals. They count upon the expressed contempt of educated physicians as capital with which to purchase the sympathies of the public. It is, therefore, a question for us to consider earnestly whether we shall meet their pretensions with that bitter hostility, which many of them actually desire, or allow them to sink quietly into obscurity. Quackery, it must be remembered, does not originate with the quack. It is the offspring of popular ignorance, and to combat it successfully we must remedy the evil from which it springs.

Faraday once delivered a lecture on the necessity of cultivating the judgment. He believed that most of the evils suffered by mankind at the hands of charlatans were due to the deficiencies of our educational systems in that respect. The memory, tastes and inventive faculties are cultivated with the greatest care, but not even in our highest institutions of learning are young men and women systematically and thoroughly taught to distinguish truth from error and falsehood. Our most intelligent classes, accordingly, though possessed of a considerable degree of refinement and general intelligence, are almost as easily imposed upon as the most ignorant members of society. A childlike belief in the infallibility of the senses as a test of truth, is characteristic of all classes. Tell even a college graduate, who has faith in some system of medicine, whose efficacy he has witnessed "with his own eyes," that "his eyes had not been educated to distinguish the truth in medical cases, that he was ignorant of the fundamental branches of medicine, and, therefore, incapable of judging of the effects of remedies," and he would laugh you to scorn. That relief followed the exhibition of a medicine is to him proof positive of the efficacy of the drug, and the correctness of the system on which the attending physician practiced, or pretended to practice. The credulity which men exhibit in accepting the most absurd statements and theories on insufficient grounds is, in itself, proof of the deficiencies of an education which has not taught them to estimate such testimony at its proper value. We cannot expect even to have the community so educated in medical matters as to enable individuals of other callings to judge of the correctness of medical opinions. Such a degree of proficiency would make physicians as a class superfluous. *This*, we may hope: that under improved systems of education, people may become convinced of their ignorance, and, with less conceit of their own knowledge of medicine, may have more confidence in that of men who have devoted their lives to its study. This conviction would not seem to require a highly cultivated judgment, and yet experience has shown that only the wiser men of the community have ever been able to recognize their own deficiencies. When children are taught in the schools to *reason* as well as to remember, when the sciences occupy as large a share of the curricula of our academies and colleges, as the classics, and the student is obliged to compare and weigh conflicting evidence, and learns the folly of a hasty judgment, we of the medical profession can hope to be esteemed according to our acquirements, and not before.—*Detroit Review.* T. A. McGraw, M. D.

